# How can dental insurance be optimized?

**DOI:** 10.1007/s10198-017-0938-y

**Published:** 2017-10-30

**Authors:** Piet Calcoen, Wynand P. M. M. van de Ven

**Affiliations:** Erasmus School of Health Policy and Management, Erasmus University Rotterdam, PO Box 1738, 3000 DR Rotterdam, The Netherlands

**Keywords:** Dental care, Optimal insurance, Deductible, Co-insurance, Insurability, Behavioral economics

## Introduction

In Europe, on average 70% of total expenditure on dental care is private expenditure and 16% of this private expenditure is covered by complementary dental insurance (CDI) [1]. However, most CDI products currently on the market in Belgium, France, Germany, and the Netherlands are not optimal because (1) they provide little protection against financial risk and do not improve access to otherwise unaffordable dental treatment (e.g., implants and crowns) and (2) moral hazard and adverse selection are not sufficiently counteracted. The suboptimal character of CDI can be explained by supply-side aspects (the limits of insurability) and demand-side aspects (behavioral economics). On the basis of these potential explanations, strategies will be drawn to optimize dental insurance.

We begin by presenting a framework for optimal insurance design, as well as the current situation of CDI in Belgium, France, Germany, and the Netherlands.

## Optimal health insurance design

Health insurance has two important advantages for the consumer, but also two disadvantages (see Table 1 below). On the one hand, health insurance reduces the financial risk for the insured and provides access to health care that would otherwise be unaffordable [2]. On the other hand, insurance increases costs due to loading costs—the administrative and other expenses of the insurer—and moral hazard. In relation to dental care, perhaps more so than in relation to other types of care, a choice is possible between cheaper basic treatments and more expensive ‘luxury’ treatments (e.g., placing a metal crown versus a porcelain crown), which may result in substantial moral hazard (both consumer- and supplier-induced moral hazard).

**Table 1:** Advantages and disadvantages of health insurance

Advantages	Disadvantages
Reduction of financial risk for the insured	Loading costs
Access to health care that would otherwise be unaffordable	Moral hazard

Optimal health insurance should maintain the advantages and reduce the disadvantages as much as possible. First, it should reduce the insured’s financial risk as far as possible. Because trivial risks lead to losses that can be borne by the insured without any noticeable burden, optimal insurance should not provide coverage for trivial risks. This avoids relatively high administrative expenses and loss settlement costs (loading costs) that are very high for these risks compared with the pure risk premium.

Secondly, optimal insurance should provide ‘access to health care that would otherwise be unaffordable’. This implies that cover limits should be avoided and expensive care should be covered as much as possible.

Third, the optimality of CDI can be increased by restricting the loading costs of dental insurance by increasing insurers’ efficiency.

Fourth, to reduce moral hazard, optimal insurance should involve cost-sharing arrangements such as deductibles and co-insurance, and apply managed care. A deductible makes the enrollee responsible for all costs up to a defined threshold. A co-insurance rate makes the enrollee responsible for a percentage of costs. Optimal insurance contracts should also have a stop-loss, a limit on out-of-pocket expenses [3].

Fifth, optimal insurance should also counteract adverse selection as much as possible. Adverse selection occurs when the insured knows more information about his expected losses than the insurer knows or uses in his premium setting and underwriting process. Adverse selection can be counteracted by selective underwriting (using medical questionnaires), risk rating (setting higher premiums for groups presenting high risk, e.g., age-related) and product differentiation (designing benefits so to attract lower risks).

The features of optimal health insurance are summarized in the first column of Table 4.

## Complementary dental insurance in Belgium, France, Germany, and the Netherlands

Complementary dental insurance (CDI) provides coverage for care that is either not covered or not fully covered by the mandatory basic insurance (MBI). In the four countries studied, private expenditure on dental care (i.e., not covered by MBI) expressed as a percentage of total expenditure on dental care ranges between 30% in Germany and 74% in the Netherlands (Table 2). CDI represents 3% (Belgium) to 55% (Netherlands) of total expenditure on dental care. Out-of-pocket expenditure on dental care is the highest in Belgium (42%) and the lowest in the Netherlands (19%).

**Table 2:** Dental care: expenditure and insurance

	Belgium	France	Germany	Netherlands
Average expenditure per person on dental care	€150	€160	€314	€179
Private expenditure (i.e., not covered by MBI)^a^	45%	65%	30%	74%
Complementary dental insurance (CDI)^a^	3%^b^	43%	5.4%	55%
Percentage of population with CDI	5%	95%	18%	62%

Source: [1]

^a^As share of total expenditure on dental care

^b^Authors’ estimate

The coverage provided by CDI is complementary to that provided by MBI, which covers 26% (the Netherlands) to 70% (Germany) of total expenditure on dental care. Table 3 provides an overview of the dental care covered by MBI in the four countries.

**Table 3:** Dental care covered by mandatory basic health insurance

	Belgium	France	Germany	Netherlands
Conservative care (e.g., fillings)	+++^a^	+++^a^	+++	+^b^
Orthodontics (e.g., braces)	+	+	++	−
Prosthetics (e.g., implants, bridges, crowns)	−	+	+	−
Periodontics (gum disease treatment)	+	+	++	−

+++: good coverage

++: medium coverage

+: low coverage

−: no coverage

^a^Extra billing is possible

^b^Conservative dental care is covered for children only (under age 18)

### No optimal dental insurance

Currently, CDI offered in Belgium, France, Germany, and the Netherlands cannot be described as ‘optimal’ (Table 4). German CDI responds best to the criteria of optimal health insurance.

**Table 4:** Complementary dental insurance: presence of features of optimal health insurance design

	Belgium	France	Germany	Netherlands
No upper limit on coverage	−	−	+	−
No coverage of trivial risks	−	−	−	−
Deductible	−	−	−	−
Co-insurance	+	−	+	+
Cap on out-of-pocket expenses	−	−	−	−
Selective underwriting	−^a^	−^a^	+	−^a^
Risk rating	+	+	+	+
Product differentiation	+	+	+	+
Managed care	−	+	+	+

−: The feature is not present in CDI products offered

+: The feature is present in CDI products offered

^a^Selective underwriting is applied only for a limited number of contracts, i.e., those with the highest upper limits on coverage

Only in Germany are there no upper limits on coverage for dental care (after an initial period). CDI in the other three countries does not provide access to otherwise unaffordable health services and protection against unpredictable high financial risks. For example, replacing four teeth by implants and crowns can cost about €10,000. Upper limits of only €250 (the Netherlands) or €1000 (Belgium) do not provide protection against high financial risks nor do they make this kind of dental care more accessible. In France, even the most extensive complementary covers provide a maximum amount of only €750 per year for implants. With total costs for an implant easily amounting to about €2500, €1750 still needs to be paid for out-of-pocket after complementary insurance has kicked in. In all four countries, CDI provides coverage of trivial risks.

In all four countries, cost sharing is applied, but only in the form of co-insurance. Co-insurance rates vary between 0 and 50% in Belgium, 0 and 55% in Germany, and 0 and 25% in the Netherlands. In France, co-insurance is generally not used. In Germany and the Netherlands, many products are offered with 0% co-insurance. Deductibles are not used. Caps on out-of-pocket expenses, which protect the consumer against high financial risk, are not applied in any of the four countries studied.

Selective underwriting is primarily used in Germany, and to a lesser extent in the other three countries. In Belgium, selective underwriting is used by only one insurer offering CDI. In France, where a 7% tax has to be paid for contracts that apply selective underwriting, most CDI contracts abstain from selective underwriting. In Germany, a medical questionnaire needs to be filled out for most CDI products. The insurer can decline to cover the candidate or charge an additional premium or exclude missing teeth or the use of certain techniques from the scope of coverage. Insurance products without selective underwriting usually have contractual clauses excluding reimbursement for problems that existed well before the start of the contract and for treatments running at the moment of the conclusion of the contract (‘pre-existing conditions’). In the Netherlands, selective underwriting is rarely applied (only for the high-end dental coverage).

In all four countries, risk rating and product differentiation are used to a certain extent (e.g., age-related premiums). Products are designed and marketed to attract certain market segments.

In all countries except Belgium, preferred provider networks are used as an element of managed care for CDI. In all countries, waiting times are used as a means to contain costs and to counteract adverse selection.

## Why is dental insurance suboptimal?

Both supply-side aspects (the limits of insurability) and demand-side aspects (behavioral economics) may explain why dental insurance is not optimal.

### Limits of insurability

A potential explanation of why insurers offer suboptimal CDI is that optimal CDI exceeds the limits of insurability. According to Berliner [4], risks properly belong in the area of insurability where: (1) losses occur with a high degree of randomness; (2) the maximum possible loss for the insurer is limited; (3) the average loss amount upon loss occurrence is small; (4) the average time interval between two loss occurrences is small (i.e., losses occur frequently); (5) the insurance premium is sufficiently high; (6) there is virtually no possibility of moral hazard; (7) coverage of the risk is consistent with public policy; and (8) the law permits the cover.

Public policy (7), moral hazard (6), the degree of randomness of losses (1) and the maximum possible loss (2) are important issues as far as the suboptimality of CDI is concerned.

Public policy plays an important role in Belgium, France, and the Netherlands, where the ‘solidarity’ principle is paramount in health care financing. Equal access to health insurance is at the heart of the values of the mutual insurers (‘mutuelles’) established in those countries. Their goal is to organize solidarity between their members for the reimbursement of health care costs. According to the French ‘Code de la mutualité’, medical questionnaires may not be used by mutuals and thus selective underwriting cannot be applied. The solidarity idea is not restricted to mutuals. In France, for instance, a 7% tax is due when selective underwriting is applied. So, commercial insurance companies are encouraged by the French government not to apply selective underwriting. In Belgium as well, the government intervenes in the organization of CDI. Premium increases for existing clients are strictly regulated. Premiums can only be adjusted in line with the consumer price index or a specific ‘medical index’ for dental care, which is calculated annually by the Ministry of Economic Affairs.

Fear of reputational damage and the wish to avoid (further) restrictive regulation being adopted may help to explain why (commercial) insurance companies in Belgium, France, and the Netherlands refrain from applying ‘hard insurance logic’ such as selective underwriting. By offering limited coverage, which is equally accessible for all citizens, insurers willingly refrain from applying private insurance logic. Rather, they apply ‘social security mechanisms’ (open enrolment, no selective underwriting, community rating). Insurance companies may be concerned that the unfettered application of insurance logic could provoke a reaction from the regulator. In a market where it is impossible or very difficult for mutuals to engage in selective underwriting, commercial insurance companies applying selective underwriting could easily be accused of ‘cherry-picking’. By sticking to social security-type mechanisms, insurance companies err on the safe side. However, insurers’ reluctance to apply private insurance logic (such as selective underwriting) and reliance on other methods (such as setting upper limits on coverage) lead to the development of suboptimal CDI products.

Moral hazard is an important problem due to the very nature of dental care. Choices among different treatment options are strongly influenced by individual consumer preferences, where aesthetic aspects often play a role. Dentists also have their preferences, which can be influenced by the consumer’s and insurer’s ‘willingness to pay’. Dental insurance can thus provide fertile ground for both consumer- and provider-induced moral hazard. Therefore, in an optimally designed scheme, insurers ought to fully invest in countermeasures. However, classic private insurance measures such as deductibles and co-insurance are not or are not fully implemented due to public policy concerns. Rather, insurers prefer to use other measures such as offering restricted coverage, setting upper limits on coverage, and not applying a cap on out-of-pocket expenditure. However, this contributes to the development of suboptimal CDI products.

The degree of randomness of losses varies between total randomness and absolute predictability. Pre-existing conditions come close to being absolutely predictable. In Belgium, France, and the Netherlands, CDI covers pre-existing conditions. Consequently, adverse selection is inadequately counteracted and a vicious circle arises whereby the insurer needs to repeatedly increase premiums to be able to continue coverage for the high risks that have subscribed the insurance policy. This leads to the development of suboptimal CDI products [5]. By comparison, in Germany, CDI is effectively limited to future, unforeseen events. It may not be a coincidence that CDI in Germany generally provides unlimited coverage, in contrast to the situation in Belgium, France, and the Netherlands.

The ‘maximum possible loss’ is also a potential explanation for the sub-optimality of CDI. Certain dental treatments, i.e., prosthetic treatments such as the replacement of multiple teeth, constitute a risk with a relatively large loss amount (more than €10,000) and a low loss frequency. Such risks can only be made insurable if the insurer is given the opportunity to build up long-term loss reserves from its premium income. However, CDI contracts can be cancelled by the insured every year. Uncertainty about the duration of the contract together with moral hazard and adverse selection may lead insurers to limit coverage, resulting in suboptimal dental insurance.

### Behavioral economics

Van Winssen et al. [6] explored potential explanations of why individuals choose suboptimal complementary health insurance. Based on key insights from behavioral economics, they discuss several factors that can have an impact on the high uptake of suboptimal insurance by consumers. For CDI, the following factors are relevant.

Factors such as liquidity constraints and debt aversion may help to explain why people buy dental insurance products that provide only limited coverage. Liquidity constraints imply that individuals do not have the means to free up (substantial) funds at a given point in time. Debt aversion stems from mental accounting theory [7] and is illustrated by individuals’ preference to prepay for consumption and to get paid for work after completion.

Ignorance and social comparison may also affect individuals’ willingness to purchase suboptimal insurance. People often do not know exactly what they are insuring themselves against by taking out complementary dental insurance (see, e.g., [8]) and they often do not know the costs of dental care that is (not) covered by their insurance. They often rely on what their peers decide [9].

## Strategies for optimizing complementary dental insurance

In many countries, MBI does not cover certain types of dental care such as prosthetic treatments (e.g., crowns, implants, and bridges) or provides only limited coverage (see, e.g., Table 3). With 70% of total expenditure on dental care being privately financed in Europe, CDI can play an essential role in the affordability and accessibility of dental care. Therefore, it is important for CDI products to respond as closely as possible to the features of optimal insurance design. However, currently, many CDI products on the market are suboptimal. The gap with optimal insurance design can be explained by both supply-side aspects and demand-side aspects. From these potential explanations, the following strategies to optimize CDI can be drawn.

First, public policy would like voluntary CDI products to provide both optimal insurance coverage and equal access to insurance. However, this is not possible because optimal insurance requires selective underwriting and risk rating (to counteract adverse selection to protect existing clients against free riders who abuse the insurance system), which is inconsistent with the principle of equal access. Therefore, policymakers should carefully decide which types of dental care are essential and ought to be covered by MBI. Dental care that is considered non-essential by policymakers and that is therefore not covered by MBI should be subject to private insurance logic. If, because of budgetary constraints, essential dental care cannot be covered by MBI, subsidization of private insurance for persons with low incomes might be an alternative to full public provision.

Second, moral hazard could be counteracted by the systematic use of deductibles and co-insurance. Standard lists of usual market prices could be compiled (as, e.g., in the Netherlands and France) and provider networks adhering to a price list could be created. Insurers should not shy away from legal action in case of excessive amounts being claimed (i.e., excessive extra billing).

Third, selective underwriting and risk rating could be used to counteract adverse selection and to protect existing clients against free riders who abuse the insurance system. Providing insurance for pre-existing conditions is incompatible with the insurance principle that only future, unforeseen risks can be covered: a burning house cannot be insured.

Fourth, applying waiting times for expensive treatments such as prosthetics and providing only limited coverage during the initial years of the contract constitute alternatives to a general limitation of coverage. For instance, in Germany, limited coverage typically applies during the first 4 years of the insurance contract.

Fifth, behavioral economics aspects such as liquidity constraints and debt aversion could be dealt with by offering a combination of optimal dental insurance in combination with a health (dental) savings account. The dental savings account could be used to finance trivial costs. In this way, CDI could be optimized and would not be tainted by attempts to also cover trivial risks. Ignorance and social comparison can be taken care of by improving the transparency of CDI products. Consumer organizations can play an important role in clarifying the market offer for the consumer.
